# Lymphangioma circumscriptum

**DOI:** 10.11604/pamj.2014.17.68.3771

**Published:** 2014-01-28

**Authors:** Maha Mael-ainin, Badredine Hassam

**Affiliations:** 1 Service de Dermatologie, CHU Ibn Sina, Université Mohamed V, Souissi, Rabat, Maroc

**Keywords:** Lymphangioma circumscriptum, lymphangiome microkystique, système lymphatique, Lymphangioma circumscriptum, microcystic lymphangioma, lymphatic system

## Image en medicine

Lymphangioma circumscriptum ou lymphangiome microkystique superficiel est une malformation lymphatique secondaire à des anomalies localisées du développement du système lymphatique. Ces malformations sont isolées ou associées à des lymphangiomes macrokystiques. Elles surviennent exceptionnellement au cours d'un syndrome angiodysplasique. Les lymphangiomes microkystiques se révèlent à la naissance ou dans la petite enfance sous forme d'une plaque infiltrée surmontée de vésicules à contenu clair ou hématique, ou sous forme d'une plaque à surface lisse ou hyperkératosique. Les lésions siègent au niveau des membres, rarement au niveau du tronc. L’évolution est stable, parfois entrecoupée par des épisodes de lymphorée, de saignement intrakystique ou de surinfection. L'histologie cutanée objective des dilatations lymphatiques épidermiques ou dermiques. L'imagerie par résonance magnétique (IRM) détermine la nature microkystique, macrokystique ou mixte du lymphangiome, précise l'extension en profondeur et le risque de compression. Le traitement des poussées repose sur les pansements adhésifs, les antalgiques et les anti-inflammatoires systémiques. Le traitement de fond repose sur la résection chirurgicale, l’éléctrocoagulation, la sclérothérapie ou le laser CO2. Devant le risque de récidive, une abstention thérapeutique peut être justifiée. Nous rapportons le cas d'un patient âgé de 14ans, sans antécédents pathologiques, qui a présenté à l’âge de deux ans des lésions vésiculeuses à contenu clair en regard de la face interne du genou droit. Le patient présente depuis une année des épisodes de suintement clair parfois hémorragique. L'histologie cutanée et l'IRM étaient compatibles avec un lymphangiome microkystique superficiel. Seul un traitement symptomatique à base de pansements adhésifs lors des poussées a été préconisé.

**Figure 1:**
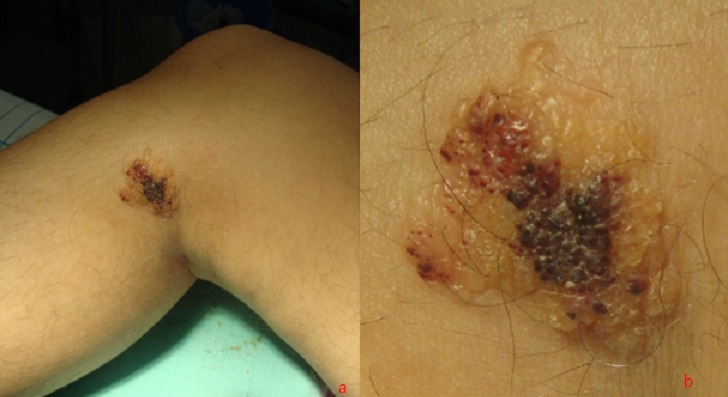
Vésicules claires et hématiques en regard de la face interne du genou droit

